# Supplementary material for: A New Approach to Imaging and Rapid Microbiome Identification for Prostate Cancer Patients Undergoing Radiotherapy
Source: Biomedicines. 2022 Jul 27;10(8):1806. doi: 10.3390/biomedicines10081806 (PMC9405325; doi:10.3390/biomedicines10081806)
Supplement: Supplementary file 1 [file biomedicines-10-01806-s001.zip › biomedicines-1817738-supplementary.pdf]

Table S1

INFORMATION FOR THE PATIENT MIDSTREAM COLLECTION FOR MICROBIOLOGICAL STUDIES
**Preparation:**<br><br>• Collect two disposable sterile containers with a cap from the Treatment Room of the Radiotherapy Unit 1.022, with a capacity of 50-100 ml described by a nurse<br><br>- before gold fiducial implantation<br><br>- on the day of starting radiotherapy before the first fraction<br><br>- on the day after the last fraction of radiation therapy<br><br>- during post-treatment control: 1 month
**Execution:**<br><br>• Before collecting the material, thoroughly wash your hands with soap and water, preferably after collecting the urine in your bladder for several hours (4 hours).<br><br>• After draining the foreskin, clean the urethral orifice thoroughly<br><br>• Take the first portion of urine into the toilet, and then, without interrupting, urinate a middle stream (approx. 5-10 ml) directly into the prepared container without touching its edges or inner surface. The rest of the urine should urinate to the toilet.<br><br>• Screw the container tightly<br><br>• Deliver urine sample to the Treatment Room of the Radiotherapy Department as soon as you urinate to container

Figure S1

(A)

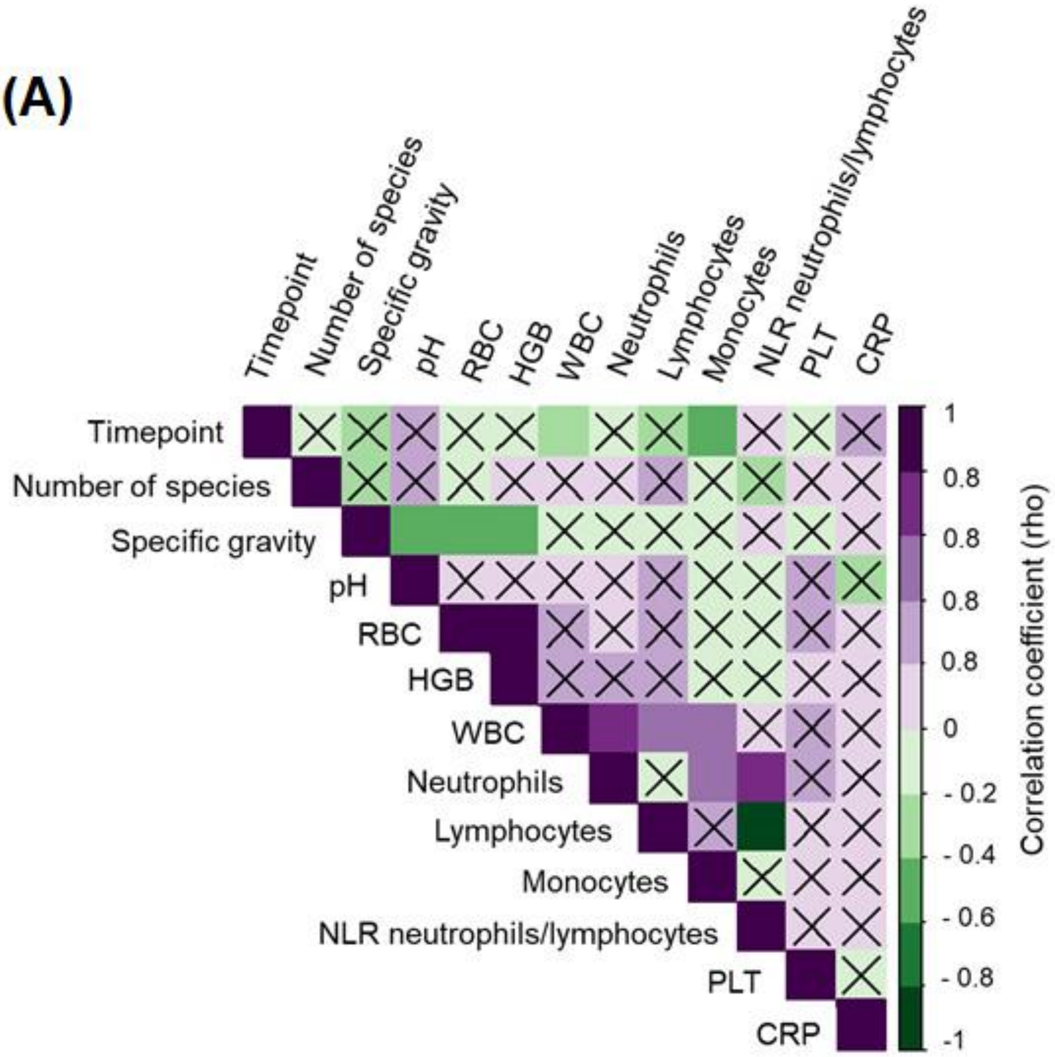

(B)

		leukocytes	number of species	specific gravity	
urine color	rho	0.297	0.210	0.419	rho
	p	0.013	0.080	0.000	p
leukocytes	rho		0.403	0.066	rho
	p		0.001	0.586	p
number of species	rho			0.020	rho
	p			0.869	p
